# Editorial for the Special Issue “The Identification of Narcotic and Psychotropic Drugs”

**DOI:** 10.3390/toxics13100852

**Published:** 2025-10-09

**Authors:** Pascale Basilicata, Maria Pieri

**Affiliations:** Department of Advanced Biomedical Science, University of Naples “Federico II”, Via S. Pansini 5, 80131 Naples, Italy; pbasilic@unina.it

## 1. Introduction

Over the last few decades, there has been a constant increase in interest in the forensic toxicology field, which has become one of the reference sciences for the elucidation of events of judicial interest [1,2]. The need to reconcile analytical problems (mainly concerning the qualitative/quantitative analysis of substances of abuse [3], particularly new psychoactive compounds [4] in complex biological matrices and seized material) with judicial requirements [5] (i.e., providing data that can be used as documentary evidence in trials [6]) makes the discipline unique among “analytical” sciences. 

The correct interpretation of analytical data [7], especially that aiming to elucidate the extent of the impairment induced by a certain substance [8,9], as well as the mechanisms regulating drugs’ toxicity [10], as well as “old” issues related to the correct interpretation of post-mortem data [11,12], represent critical aspects of research and debate among forensic toxicologists. 

This Special Issue addresses several key aspects of forensic and clinical toxicology related to the analysis of substances of abuse and psychoactive drugs in different matrices (both biological and non-biological), as well as the importance of studies on animal models to extrapolate drugs’ toxicity toward humans. 

## 2. An Overview of Published Articles

The manuscript by Tang and colleagues [13] presents the neurotoxic consequences of ketamine exposure in zebrafish. The study integrated metabolic profiling and behavioural analysis, highlighting a correlation between the extent of ketamine exposure and the degree of locomotor activity. Metabolic profiling allowed for the elucidation of similarities with humans, confirming that zebrafish could be a valid candidate as a model organism to predict ketamine toxicity. 

The research of Stern and colleagues [14] is focused on a critical tool in forensic toxicology: the prompt identification of new psychoactive compounds in street drugs. In particular, the authors focused on the identification of xylazine, used as an adulterant in the production of fentanyl and its derivatives. The analytical approach involves the use of an electrochemical sensor designed for xylazine and suitable for on-site tests performed also by non-specialized technicians.

The contribution from Dunn and colleagues [15] reports a descriptive pharmacovigilance analysis on adverse events due to drug abuse based on the Food and Drug Administration’s Adverse Events Reporting System. The results highlight increasing misuse of veterinary medications (including active principles authorized exclusively in a veterinary context), frequently present as adulterants in the illicit drug market.

The influence of storage conditions on post-mortem stability is presented by Gariglio and colleagues [16], who focused on spontaneous post-mortem methemoglibin formation. The article focuses on a critical issue in post-mortem practice, especially when death is caused by nitrite and/or nitrate poisoning. The results of both a retrospective study related to judicial cases analyzed in the period 2018-2021 and of an experimental study confirm the need for an analysis of the accuracy of the obtained results.

Zellner and colleagues’ [17] contribution focuses on the value of toxicological data in clinical practice. The authors present the results of a retrospective study on more than eight hundred patients that underwent urinary toxicological analysis after hospitalization at the Division of Clinical Toxicology of the Poison Centre of Munich in the period 2014-2022. The results propose as evidence the crucial role of performing toxicological analyses for a correct diagnosis, since anamnestic datum alone can be inaccurate due to patients frequently underreporting ingesting drugs.

Sosa’s article [18] focuses on the use of ocular surface fluid as an alternative matrix in post-mortem analysis. A survey of scholarly sources available is presented with the aim of shedding light on the potential of such a matrix for external drug screening as well as systemic disease tests.

## Data Availability

Not applicable.

